# Introducing the Y-chromosomal Ancestral-like Reference Sequence—Improving the Capture of Human Evolutionary Information

**DOI:** 10.1093/molbev/msaf222

**Published:** 2025-09-12

**Authors:** Zehra Köksal, Annina Preussner, Jaakko Leinonen, Taru Tukiainen

**Affiliations:** Department of Biomedical and Clinical Sciences, Linköping University, Linköping, Sweden; Section of Forensic Genetics, Department of Forensic Medicine, Faculty of Health and Medical Sciences, University of Copenhagen, Copenhagen, Denmark; Institute for Molecular Medicine Finland (FIMM), HiLIFE, University of Helsinki, Helsinki, Finland; Institute for Molecular Medicine Finland (FIMM), HiLIFE, University of Helsinki, Helsinki, Finland; Center for Child, Adolescent, and Maternal Health Research, Faculty of Medicine and Health Technology, Tampere University, Tampere, Finland; Institute for Molecular Medicine Finland (FIMM), HiLIFE, University of Helsinki, Helsinki, Finland; Department of Psychology, Faculty of Medicine, University of Helsinki, Helsinki, Finland; Institute of Clinical Medicine, Faculty of Health Sciences, University of Eastern Finland, Kuopio, Finland

**Keywords:** Y-ARS, ancestral state reconstruction, chrY, Y-chromosomal haplogroups, derived state, polaryzer

## Abstract

Reference sequences are essential for reproducible genetic analyses but are often chosen without regard to evolutionary relevance within the analyzed species. The human Y chromosome is widely used in evolutionary studies, yet current references represent evolutionarily young sequences, which can cause misleading variant calling. To address this issue, we constructed a Y-chromosomal ancestral-like reference sequence to improve the detection of evolutionarily informative variants on the Y chromosome. The Y-chromosomal ancestral-like reference sequence was constructed by applying a weighted maximum parsimony approach to human and primate Y chromosome sequences. To benchmark the performance of the Y-chromosomal ancestral-like reference sequence, 40 Y chromosome short-read sequences from diverse haplogroups were aligned to Y-chromosomal ancestral-like reference sequence and existing references (GRCh37, GRCh38, and T2T-CHM13). Overall, the Y-chromosomal ancestral-like reference sequence yielded the highest and most consistent number of SNPs per sample (mean = 1,400; SD = 77), while other references yielded on average fewer variants (mean = 866 to 968) and showed greater variability across samples (SD = 457 to 531) depending on their phylogenetic distance from the reference. Additionally, alignments to the Y-chromosomal ancestral-like reference sequence resulted in calling solely SNPs with evolutionarily derived alleles, while alignments to other references resulted in calling on average 46% SNPs with ancestral alleles. This study demonstrates how the existing reference sequences fail to capture the full range of evolutionary information on the Y chromosome. The Y-chromosomal ancestral-like reference sequence improves capturing evolutionary information on the Y chromosome, making it a valuable resource for various evolutionary applications, such as TMRCA estimations and phylogenetic analyses. Finally, alongside the Y-chromosomal ancestral-like reference sequence, we provide a publicly available tool, polaryzer, to annotate variants as ancestral or derived in pre-aligned Y chromosome data.

Significance Using current reference sequences results in calling genetic variants without information on whether the variant is ancestral or arose later in the species' history, which is of interest for evolutionary studies. Here, we tackle this problem for the human Y chromosome by introducing the Y-chromosomal ancestral-like reference sequence, Y-ARS. The Y-ARS overcomes this issue by calling only evolutionarily derived variants on the Y chromosome in a reproducible way, which can directly be used for various downstream analyses in evolutionary genetics.

## Introduction

Reference sequences are essential for reproducible genetic analyses, as they establish a standard sequence and an universal coordinate system, enabling consistent gene annotation, functional analysis, and representation of genetic diversity in the form of genetic variants (Worley et al. 2017; Wong et al. 2020). The reference genomes of well-studied organisms, such as the human or mouse, are consistently improved by applying newer sequencing technologies and providing more complete references (Nurk et al. 2022). Whereas the classical reference sequences, such as human GRCh37 (GCF_000001405.25, NCBI Assembly), GRCh38 (GCF_000001405.40, NCBI Assembly), and T2T-CHM13 (T2T) (Nurk et al. 2022) are largely derived from only one contemporary sample, emerging pangenomes based on multiple samples contain more within-species diversity (Liao et al. 2023). However, all the current reference sequences lack information on evolutionary states of the variants, which is fundamental information for evolutionary studies.

In evolutionary and population genetic studies, the Y chromosome (chrY) is one of the widely utilized sequences. Since the MSY (male-specific region of chrY) escapes recombination, all of its genetic variations are inherited together as a haplotype, making it a useful tool for tracing back mutational changes for the study of demographic events. These Y-chromosomal haplotypes can be classified into haplogroups (named alphabetically from A to T) that share a common ancestor. Although haplogroup A is the oldest lineage, diverged from the rest at around 160 to 307 kya (Karmin et al. 2015; Hallast et al. 2023), Y chromosomes of the current human genome references are derived mostly from evolutionarily young haplogroups R1b (GRCh37, GRCh38) and J1 (T2T), formed approximately 15 to 26 kya and 13 to 24 kya, respectively (Karmin et al. 2015; Hallast et al. 2023).

Using an evolutionarily young reference is problematic for evolutionary and population genetics studies. On the one hand, all alleles that are identical between the sample and the reference are disregarded as these emerge as nonpolymorphic in variant calling, although some of these might contain evolutionarily relevant alleles. On the other hand, evolutionarily ancestral alleles might be called as sequence variants at sites where the reference contains a derived allele. As a result, the identified variants lack information on the allele polarization, i.e. whether the observed alleles are evolutionarily ancestral or derived. Instead, the called variants only represent differences between the sample and the reference genome. Yet, several applications, such as assessing sequence evolution, phylogenetic analyses, and demographic modeling, currently rely on knowledge of the evolutionary states of (Y-chromosomal) alleles (Kelleher et al. 2019; Speidel et al. 2019; Köksal et al. 2023).

For individual genomic sites, the ancestral states of nucleotides can be reconstructed using methods applying maximum parsimony (Collins et al. 1994) (e.g. MEGA; Tamura et al. 2021) or maximum likelihood (Felsenstein 1981; Koshi and Goldstein 1996) (e.g. PAML and FastML; Ashkenazy et al. 2012). For individual sites on the human chrY, the ancestral states are usually reconstructed by comparing the samples to an evolutionary outgroup, such as primates (Hallast et al. 2015; Karmin et al. 2015). However, currently there is no easily applicable, streamlined and reproducible solution to aid in determining evolutionarily derived alleles on the human chrY in a straightforward manner.

In this study, we constructed an ancestral-like reference sequence for the human Y chromosome (Y-ARS), to provide a reproducible solution to call only evolutionarily derived variants on the chrY. To construct the Y-ARS, we applied weighted maximum parsimony (WMP) to determine ancestral alleles in the T2T chrY, using eight long-read human sequences of diverse major Y-chromosomal haplogroups for increased sequence overlaps and capture of human diversity. As evolutionary outgroups in the WMP, the four long-read primate sequences (chimpanzee, bonobo, gorilla, orangutan) were used. We benchmarked the Y-ARS with 40 short-read human sequences from most major haplogroups (A to T), which were aligned to the Y-ARS and existing references GRCh37, GRCh38, and T2T. To enable the use of Y-ARS beyond alignment-based applications (i.e. when handling variant calling format [VCF] data), we developed polaryzer, a tool that annotates called variants from other references as ancestral or derived. The novel Y-ARS reference sequence, as well as polaryzer, are made publicly available to simplify the process of determining evolutionarily derived alleles on the chrY in a reproducible way.

## Results

### The Y-ARS Shows Advantages in Annotating Ancestral States Over SNP Databases

**Fig. 1.**
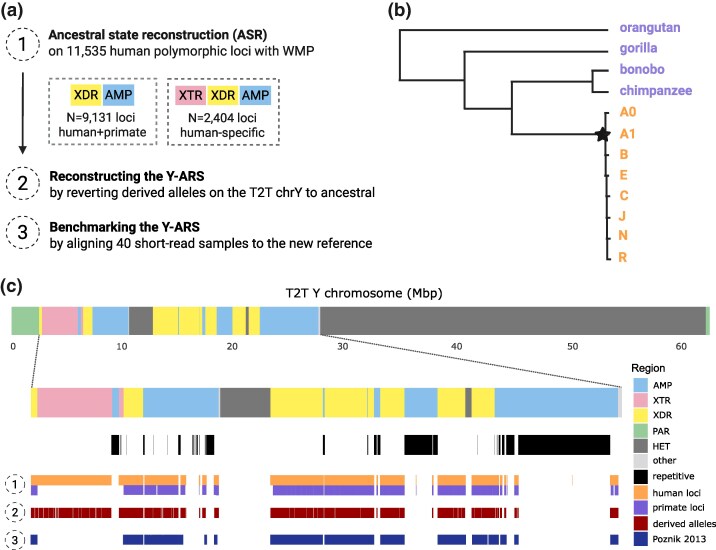
Overview of the a) study workflow, b) underlying phylogenetic relationships for ASR with the focal node (marked with a star), and c) locations of considered loci at each part of the study on the T2T chrY, annotated as AMP, XTR, XDR, pseudoautosomal (PAR), heterochromatin (HET), and other. The overall length of the chrY considered for ASR on the XDR, XTR, and AMP is 15.6 Mbp, excluding highly repetitive regions (i.e. palindromes, TSPY, and inverted repeats) marked in black. The study was conducted in three main steps. (1) First, we reconstructed ancestral alleles using WMP at 11,535 human-polymorphic loci (shown in top panel [orange]), comprising 9,131 loci shared with primates (shown in bottom panel [purple]), and 2,404 human-specific loci (including the XTR). (2) To construct the Y-ARS, 2,545 alleles identified as evolutionarily derived on the T2T chrY were converted into ancestral alleles (shown in red). (3) Finally, to evaluate the Y-ARS, 40 short-read sequences were aligned to the Y-ARS on 10.5 Mbp of the chrY defined as accessible for short-read sequencing, as defined by Poznik et al. (2013) (shown in dark blue).

To obtain the Y-ARS, we began by assessing sites that are polymorphic among humans within the nonrepetitive regions of the chrY (∼15.6 million bp) (Fig. 1). At each polymorphic site identified (*N* = 11,535 among the eight human sequences), we applied a WMP approach to infer the most probable ancestral allele using data from eight human and four primate Y chromosomes (Fig. 1). To gain the Y-ARS, we then converted all alleles identified as evolutionarily derived on the T2T chrY (*N* = 2,545) back to their ancestral state (Supplementary table S1, Supplementary Material online). This number represents the SNPs that have accumulated on the T2T chrY since its divergence from the most recent common ancestor (MRCA), i.e. the Y-ARS. This places the age of the Y-ARS at approximately 187 kya (CI 173 to 203 kya), which is in line with previous estimates dating the MRCA of the human chrY to 160 to 307 kya (Karmin et al. 2015; Hallast et al. 2023).

To validate the ancestral states of the alleles in the Y-ARS, we accessed SNP entries in the human Y-SNP database YBrowse (https://ybrowse.org/). YBrowse is a comprehensive database of haplogroup-defining Y-chromosomal SNPs, which includes information on the ancestral and derived alleles of each SNP submitted by individual researchers. The ancestral alleles of SNPs in the YBrowse database were compared with the alleles in the Y-ARS of the corresponding Y-chromosomal sites. Along the Y-chromosomal area of interest (15.6 Mb), we identified in total 1,455,104 sites with an existing YBrowse annotation. These corresponded to 1,563,166 SNP entries, given some sites had several database entries. For the vast majority of the identified sites (99.6%; *N* = 1,448,699), the allele observed in the Y-ARS corresponded to the ancestral allele in the YBrowse database, supporting the validity of the ancestral state of the Y-ARS alleles.

However, for a small fraction of the sites (0.6%; *N* = 8,975), the allele in the Y-ARS carried the derived allele (i.e. a contradiction) according to the YBrowse database. Yet, most of these sites (*N* = 7,492) were not polymorphic among our data, possibly characterizing (sub)haplogroups not represented by the eight samples used to reconstruct the Y-ARS in the current study. Alternatively, these could be possible errors in the database caused by allele switch-ups between the ancestral and derived allele. For the sites that were polymorphic among our data (*N* = 1,483), we assessed these contradictions in more detail (Supplementary figs S1 and S2, Supplementary Material online). A considerable fraction of these contradicting annotations (*N* = 1,210) occurred at sites with multiple annotations with different alleles reported as the ancestral allele (Supplementary fig. S2a, Supplementary Material online). Here, back-mutations (i.e. two mutational events at the same genomic site occurring by chance) appeared to account for many of the overlapping annotations (Supplementary fig. S2Aa, Supplementary Material online). Some of the sites with multiple database entries were not necessarily explainable by back-mutations, but showed support for the observed Y-ARS allele being ancestral (Supplementary fig. S2Ab and S2Ac, Supplementary Material online). A small portion of the sites with contradicting annotations did not have multiple annotations, but were annotated only as derived in the database (*N* = 273) (Supplementary table S2, Supplementary Material online). Yet, on all of these sites our data supported the Y-ARS allele to be ancestral (Supplementary fig. S2b, Supplementary Material online). For a small number of sites (*N* = 33) we could not infer the ancestral alleles with high confidence, due to high mutability (Supplementary fig. S2Bg, Supplementary Material online) or high rates of missingness (Supplementary fig. S2Bd,f, Table S2, Supplementary Material online). Altogether, examining these contradicting annotations showed that the Y-ARS provided more often reliable annotations for Y-chromosomal SNPs than the YBrowse database on the compared sites. Such database contradictions might be caused by submissions from individual studies or testing groups, rather than comprehensive population-wide data or phylogenetic reconstructions.

To investigate the impact of evolutionary conservation of a site on the reliability of the 11,535 reconstructed alleles, we analyzed loci classified as highly conserved by Swiel et al. (2025). This classification is based on the criteria of the loci being (i) uniquely mapping with short-read sequencing data and (ii) conserved on the chimpanzee chrY. To quantify the reliability of the reconstructed alleles, we calculated an uncertainty score for each of the 11,535 reconstructed ancestral sites (Supplementary fig. S3, Supplementary Material online), where scores closer to 0 indicate higher confidence of the reconstructed allele (see Materials and Methods). Overall, we observed that the alleles located on evolutionarily conserved regions (*N* = 4,703) had significantly lower uncertainty scores when compared to nonconserved regions (*t*-test for loci classified as uniquely mappable with short-read data *P* = 3.59e to 104; and for chimpanzee conserved loci *P* = 6.01e to 177) (Supplementary fig. S4, Supplementary Material online). However, more than 95% of alleles located outside uniquely mappable and primate-conserved regions also provided reliable ancestral annotations (i.e. defined as uncertainty scores below 0.25; see Supplementary table S3, Supplementary Material online) in the ancestral state reconstruction (ASR), highlighting that (complete) primate conservation is not necessary to reconstruct the ancestral alleles in humans. Accordingly, the majority of alleles on the Y-ARS appeared to be confidently ancestral regardless of their evolutionary conservation (uncertainty score average = 0.117; median = 0.100; SD = 0.049) (Supplementary fig. S3, Supplementary Material online).

Nevertheless, a key challenge in ASR remains in accurately determining ancestral alleles in samples that are phylogenetically close to the focal node. (i.e. haplogroup A0). As the focal node is located right before the A0 haplogroup, it becomes difficult to infer whether the allele carried by A0 has formed before or after the A0 haplogroup split from the MRCA of the human chrY. To evaluate this possible haplogroup A-bias within the Y-ARS, we further extracted all SNPs, where an allele was carried exclusively by the sample of haplogroup A0 among humans. We identified 2,520 of such sites (Supplementary table S4, Supplementary Material online), which we further explored in additional human sequences of haplogroup A0 and A00 and primate data (see Materials and Methods). While the majority of alleles at these sites (88%; *N* = 2,210) supported the Y-ARS allele being the ancestral allele, all sites could not be conclusively re-evaluated, resulting in the true ancestral allele remaining ambiguous on 310 sites, listed in Supplementary table S4, Supplementary Material online.

### Benchmarking the Y-ARS Using Short-read Data Underlines its Advantages in Calling a Balanced Number of Evolutionarily Derived SNPs

**Fig. 2.**
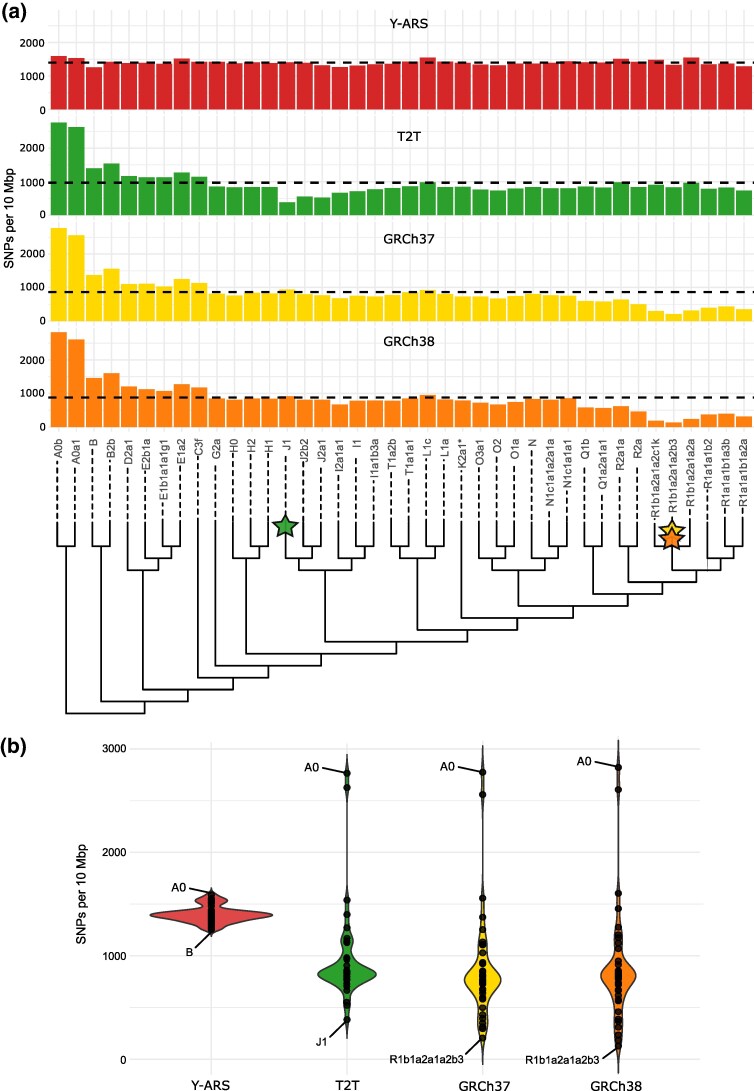
a) Number of chrY SNPs normalized by per-sample coverage (and multiplied by 10 million) per reference across all samples, spanning the majority of known Y-chromosomal lineages (see full phylogeny in Supplementary fig. S6, Supplementary Material online). Phylogenetic location of references T2T, GRCh37, and GRCh38 representing downstream haplogroups are marked with stars on the phylogenetic tree. References GRCh37 and GRCh38 represent haplogroup R1b1a1b1a1 and T2T represents haplogroup J1a2a1a2c1a1. The dashed lines indicate the mean SNPs over all samples for each reference. b) Variability in SNP counts on each reference sequence for all samples. Samples with the lowest and highest number of SNPs observed for each reference are annotated.

To quantify how the Y-ARS impacts variant calling on the chrY, we aligned 40 short-read human chrY sequences from most major haplogroups (A to T) to the Y-ARS and existing references GRCh37, GRCh38, and T2T. After quality filtering and excluding poorly mappable regions (Fig. 1; Supplementary fig. S5, Supplementary Material online), the Y-ARS resulted in calling the highest number of SNPs when averaged across all samples (*N* = 1,400 normalized SNP count per 10 Mbp), compared to GRCh37 (*N* = 866), GRCh38 (*N* = 878) and T2T (*N* = 968) (Fig. 2a). Furthermore, the number of SNPs called upon alignment to the Y-ARS were relatively constant across samples (standard deviation SD = 77; range = 1,029 to 1,585), while the GRCh37 (SD = 505; range = 206 to 2,774), GRCh38 (SD = 531; range = 129 to 2,821), T2T (SD = 457; range = 383 to 2,765) displayed more variation in the total number of SNPs called (Levene's test *P* = 0.012) (Fig. 2a and b).

For the three standard reference sequences, the number of SNPs called was lower in cases where the reference sequence and the sample sequence belonged to phylogenetically close haplogroups (Supplementary table S5, Supplementary Material online; Fig. 2a and b). Aligning to the T2T reference, which represents a haplogroup J1 sequence, resulted in calling the smallest number of variants for sample HG01253 of haplogroup J1 (*N* = 383) compared to other samples aligned to the same reference (*N* = 968 mean; *N* = 836 median across all samples). Similarly, alignments to references GRCh37 and GRCh38, which both represent haplogroup R1b sequences, resulted in calling fewer variants for samples of haplogroup R1b compared to samples of other haplogroups. For instance, sample NA19652 of haplogroup R1b1a2a1a2b3 resulted in calling six times fewer SNPs on GRCh38 (*N* = 129) compared to other samples aligned to the same reference (*N* = 878 mean; *N* = 808 median across all samples). Accordingly, the number of SNPs increased when the sample and the reference belonged to phylogenetically distant haplogroups. Generally, most SNPs were called for the samples among the oldest and most basal branches. Accordingly, samples of haplogroup A0 carried up to three times more SNPs when aligned to GRCh37, GRCh38, and T2T compared to other samples aligned to the same references (Fig. 2).

### Evolutionarily Young References Result in Calling a Mixture of Ancestral and Derived Alleles

Since the Y-ARS is an ancestral sequence to most human chrYs, all SNPs called upon alignment to Y-ARS are expected to be evolutionarily derived. However, since references GRCh37, GRCh38, and T2T are evolutionarily young haplogroups, samples aligned to these result in calling SNPs, which can be from an evolutionary perspective derived or ancestral. To determine the evolutionary state (i.e. the polarization) of alleles upon alignment to GRCh37, GRCh38, and T2T, we annotated alleles that match with the Y-ARS allele as ancestral and alleles that deviate from the Y-ARS as derived, using a custom-made tool “polaryzer” (accessible on Github; see Data Availability). When polarizing the variants of the 40 samples, we first lifted alignments from GRCh37 and GRCh38 to T2T coordinates to exclude variants without information for polarization (Fig. S7, Supplementary Material online).

**Fig. 3.**
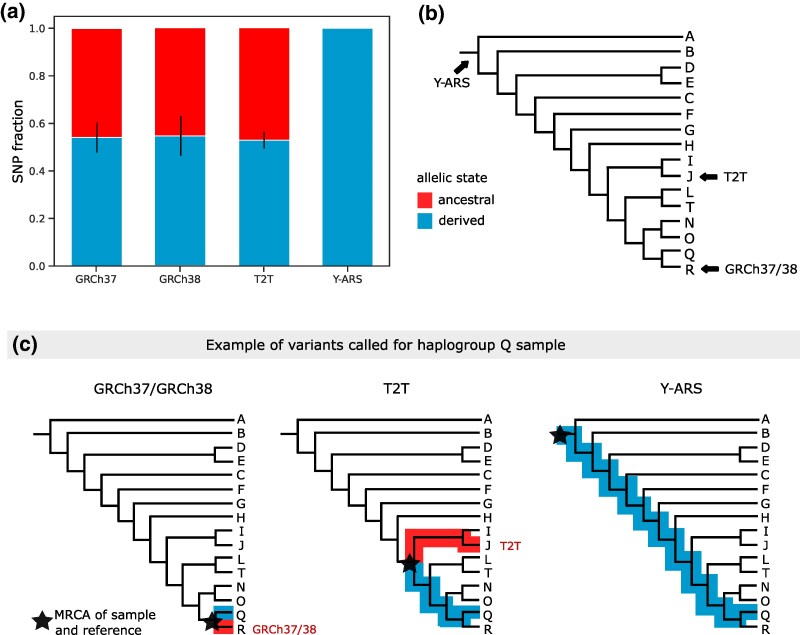
a) Average fractions of evolutionarily derived (bottom bar in blue) and ancestral (top bar in red) alleles of variants observed across all 40 samples after alignment to each reference sequence using software polaryzer. The bar height represents averages over all samples, and the error bars the standard deviations. b) Indication of phylogenetic locations of each reference sequence. c) Example breakdown of SNPs captured for haplogroup Q sample upon alignment to references GRCh37/GRCh38, T2T and Y-ARS, divided into evolutionarily derived in the reference (in red) and evolutionarily derived in the sample (in blue). Variants that are evolutionarily derived in the reference appear as ancestral alleles in variant calling, as illustrated in panel A. Only SNPs formed after the MRCA of the sample and the reference are captured.

When considering the evolutionary states of the called variants upon alignment to GRCh37, GRCh38, and T2T, on average 46% of the called SNPs represented evolutionarily ancestral alleles (Fig. 3a). The average proportions were fairly similar across these references: 46% on GRCh37 (*N* = 331) (range = 21% to 53%; 46 to 1,404 SNPs), 45% on GRCh38 (*N* = 317) (range = 13% to 53%; 21 to 1,366 SNPs) and 47% on T2T (*N* = 371) (range = 36% to 53%; 87 to 1,417 SNPs) (Fig. 3a; Supplementary fig. S8, Supplementary Material online). The remaining 54% of the called SNPs represented evolutionarily derived alleles on these references. In contrast, all SNPs called on Y-ARS-aligned samples represented evolutionarily derived variants, formed after the MRCA of the human chrY (Fig. 3, Supplementary material online).

To thoroughly characterize the observed SNPs on each of the references beyond the ancestral and derived states, we assessed how many of these had an existing annotation in SNP databases (dbSNP, ISOGG, YFull). The dbSNP-annotated sites represent known genetic variants across populations, while the ISOGG and YFull annotations highlight phylogenetically informative SNPs on the chrY, which are commonly used in haplogroup classification and chrY phylogenetic analyses. Thus, sites that lack these annotations likely lack a phylogenetic context or are private or rare SNPs in the sample or the reference.

When aligned to GRCh37, GRCh38, and T2T, on average 84% of the called SNPs had an existing annotation (48% in dbSNP, 69% in ISOGG/YFull, 33% in both) (Supplementary fig. S9, Supplementary Material online). Upon alignment to the Y-ARS, the average percentage of annotated variants was 95% (89% in dbSNP; 70% in ISOGG/YFull, 65% in both) (Supplementary fig. S9, Supplementary Material online). This was anticipated given the greater phylogenetic distance between the samples and the Y-ARS compared to other references, which allowed detecting SNPs that arose earlier in the phylogenetic tree. The proportion of haplogroup-defining SNPs on Y-ARS-aligned samples (average 70%) was similar to that of the other references (average 69%). However, samples aligned to the Y-ARS carried 1.9-fold more haplogroup-defining alleles, since all evolutionarily derived alleles were present in the sample (rather than in the reference sequence) (Fig. S10, Supplementary Material online).

When examining whether the proportion of annotated variants differed across samples aligned to the same reference, all haplogroup R1b samples (HG01785, NA19652, and HG00096) were outliers for references GRCh37 and GRCh38 (Supplementary fig. S10, Supplementary Material online). These samples showed a notably lower proportion of annotated SNPs upon alignment to GRCh37 and GRCh38 (average 33%) compared to other samples aligned to the same references (average 83%) (Supplementary fig. S9, Supplementary Material online). This trend was anticipated, as a more recent split between the haplogroups of the sample and reference allows for fewer mutations to accumulate in the sample that are absent in the reference. In these cases, private mutations (i.e. nonannotated variants) accounted for a large fraction of the total variants. Intriguingly, haplogroup J1 sample (HG01253) did not show a similar decrease in the proportion of annotated SNPs when aligned to the T2T reference (average 72%) compared to other samples aligned to the same reference (average 86%) (Supplementary fig. S9, Supplementary Material online). A possible explanation could be the larger phylogenetic distance between sample J1 and the T2T reference compared to R1b samples and the GRCh37 and GRCh38 references. This was supported by the total number of SNPs observed, as HG01253 (haplogroup J1) and T2T differed in *N* = 383 SNPs, while NA19652 (haplogroup R1b1a2a1a2b3) and GRCh38 differed only in *N* = 129 SNPs (Fig. 2a).

As expected, the alignment to the Y-ARS resulted in calling only a few haplogroup-defining SNPs per sample with ancestral alleles (median *N* = 17). These annotations were mostly characteristic of haplogroup A0. As we described before, these haplogroup A-defining SNPs likely contain annotation errors in the haplogroup databases rather than wrongly reconstructed alleles on the Y-ARS (Supplementary fig. S1, Supplementary Material online; see example in Supplementary fig. S2Bh, Supplementary Material online) deriving from the close proximity of haplogroup A to the focal node.

## Discussion

Although current reference sequences, such as GRCh37 and GRCh38, have been used for more than a decade as a standard tool in genetic research, the effect of their evolutionary state on variant calling has not been considered thoroughly. While some efforts have been made to improve the study of mitochondrial DNA (Behar et al. 2012), the chrY remains largely unassessed in this context. To overcome this issue, we constructed an ancestral-like reference sequence for the chrY, using WMP for SNPs on ∼15.6 Mbp of nonrepetitive regions of the chrY. The reconstructed Y-ARS, based on eight human and four primate sequences, represents the most recent common ancestral sequence to the majority of known human paternal lineages.

Our validations of the Y-ARS showed that the number of sites (*N* = 2,545) at which we reverted the T2T template allele to the ancestral state, was in accordance with expectations given an average Y-chromosomal mutation rate. When evaluating the Y-ARS alleles using the comprehensive database of Y-chromosomal SNPs, YBrowse, we observed that the majority (99.6%) of alleles on the Y-ARS were supported by existing database information. Only for a small portion of alleles on the Y-ARS (0.4%), we observed solely contradicting database annotations, which were in most cases providing a more reliable annotation in our data compared to the database. These contradictions could be explained for instance by back-mutations or mislabeling of reference and ancestral alleles. The latter was particularly noticeable for SNPs defining haplogroup R, which is the haplogroup of the GRCh37 and GRCh38. Although the database information was intended to validate the ancestral states of alleles in the Y-ARS, the Y-ARS turned out to provide more reliable ancestral annotations on several sites. The strength of the ASR approach is that it relies on comprehensive phylogenetic assessment and systematic cross-species comparison, while database annotations originate mostly from independent submissions from specific study cohorts or genetic testing groups, with limited standardization or evaluation of the submissions. These findings strongly underline that caution is needed when relying solely on any database annotations. The Y-ARS provides additional advantages in determining the allele polarization of SNPs not yet reported in databases with a phylogenetic context.

Although the Y-ARS showed high confidence for the majority of alleles to be ancestral, a few sites could not be reconstructed confidently. These included sites with high mutability or sites with only a few data points of the human and primate samples during ASR, which can generally be considered as evolutionarily less conserved sites. When exploring sites classified as highly conserved on the chrY by Swiel et al. (2025), we found that the reconstructed ancestral alleles were generally reliable even at sites with lower evolutionary conservation, such as sites lacking primate data (Supplementary table S3, Supplementary Material online). Nevertheless, available primate data generally provided additional support at highly mutable sites with limited number of detected alleles across samples. Despite leveraging the cross-species dataset for the ASR, inferring the ancestral alleles remained challenging at sites with haplogroup A0-specific alleles due to the haplogroup's proximity to the focal node. Even when including additional data from samples of haplogroup A0 and A00, the ancestral state remained ambiguous for sites that did not show variation beyond this haplogroup. Thus, it is possible that the Y-ARS could contain alleles that are in fact specific to haplogroup A (Supplementary table S4, Supplementary Material online). In particular, this may be reflected in the slightly increased total number of variants called for haplogroup A0 samples upon alignment to the Y-ARS (*N* > 1,500), compared to the average number of SNPs observed across all samples when aligning to the Y-ARS (*N* = 1,400). These sites with putative A0-specific alleles, along with other lower-confidence variants, have been flagged in Supplementary table S3, Supplementary Material online to enable their exclusion from downstream analyses, if necessary. Nevertheless, a slight increase in SNPs in samples of haplogroup A could also be attributed to the fact that haplogroup A has the most basal Y-chromosomal lineages with earliest divergencies from other chrY lineages approximately 160 to 307 kya (Mendez et al. 2013; Scozzari et al. 2014; Karmin et al. 2015; Barbieri et al. 2016; Hallast et al. 2023), leading lineages to undergo a longer period of branch-specific evolution.

To benchmark the use of the Y-ARS as a reference sequence, we further aligned short-read sequencing data of 40 samples to the Y-ARS and existing references GRCh37, GRCh38, and T2T. When evaluating the Y-ARS in chrY sequence alignment and variant calling, it displayed advantages compared to existing references. Aligning samples to the Y-ARS resulted in consistent SNP calling across different haplogroups (range = 1,255 to 1,589) of contemporary sequences, due to calling all SNPs formed since the MRCA of the chrY. Samples aligned to the other references (GRCh37, GRCh38, T2T), showed more variability in the number of SNPs called (range = 129 to 2,821), depending on the phylogenetic location of the sample in comparison to the reference. This variation results from calling SNPs formed after the split of the reference and sample haplogroups, while evolutionarily derived SNPs emerged before the split appear as monomorphic. The most drastic decrease in the number of SNPs (up to 6-fold) was observed for haplogroup R1b samples when aligning these to GRCh37 and GRCh38, since these samples and references share a very recent common ancestor (11 to 29 kya estimated from our data).

In addition to providing consistent SNP calling across haplogroups, the Y-ARS resulted in calling only evolutionarily derived SNPs, while the other references resulted in calling a mixture of evolutionarily derived (54% on average) and ancestral (46% on average) variants. These evolutionarily ancestral variants reflect sites where the reference sequence carries a derived allele, and such variants are therefore redundant for analyses focusing on the sample of interest. A similar pattern was also evident in the number of known haplogroup-defining alleles identified in the samples, which was nearly twice as high when using the Y-ARS reference (70%) compared to other references (37%). We observed that the number of haplogroup-defining alleles decreased (down to 3% to 21%) in particular for samples of haplogroup R1b aligned to GRCh37 and GRCh38, due to the increased capture of private or rare mutations for these samples. This highlights that aligning sequences to a phylogenetically close reference sequence results not only in calling fewer variants, but also significantly decreases the proportion of phylogenetically informative variants captured. Given that haplogroup R1b is relatively common across Europe (Myres et al. 2011), the loss of information could be concerning for variant studies on chrY data of European origin when aligning to GRCh37 or GRCh38.

While we emphasize the strengths of the Y-ARS for chrY sequence alignment and ancestral state annotation, it is important to acknowledge its current limitations. At present, the ancestral states annotated on the Y-ARS are limited to single nucleotide variations among the 15.6 Mb of nonrepetitive DNA sequence. These are the most commonly used genetic variants in chrY research, yet they represent only a subset of the potential genomic variations. For studies focusing on (the evolution of) structural variants, it could be worthwhile in the future to examine an ancestral sequence that also includes insertions and deletions (indels) and structural variants (outside of palindromic, repetitive regions), or a pangenome, although the repetitive nature of the chrY might impede these attempts.

In essence, the Y-ARS enables calling of solely evolutionarily derived variants, whereas alternative references demand manual variant annotation, which is time-consuming, error-prone, and lacks a standardized pipeline. Understanding the evolutionary state of Y-chromosomal variants is crucial for several downstream analyses and applications in chrY research, including for instance sequence age estimation (Poznik et al. 2013; Solé-Morata et al. 2017), refined phylogenetic classification of variants and haplogroups (Hallast et al. 2015; Köksal et al. 2024), and demographic modeling (Batini et al. 2015; Sahakyan et al. 2021). Particularly, when investigating novel SNPs to increase the resolution of the Y-chromosomal haplogroups, identifying the evolutionarily derived SNPs, their phylogenetic hierarchy and geographic distribution can shed light on potentially unexplored prehistoric and historic migration pathways.

Overall, this new reference sequence represents the estimated ancestral states for Y-chromosomal SNPs on the MRCA of most known human male lineages, allowing for consistent variant calling across different haplogroups. The Y-ARS can be seamlessly integrated into existing pipelines of chrY sequence alignment. To enhance the broader usability of the Y-ARS, we also offer a tool, polaryzer, to annotate the evolutionary state of alleles on pre-existing VCF files including uncertainty scores for each locus representative of the confidence of the Y-ARS allele. In conclusion, the new Y-ARS provides a consistent and reproducible way for identifying evolutionarily derived variants on the human chrY.

## Conclusion

This study demonstrates how current references (GRCh37, GRCh38, T2T) fail to capture the full range of evolutionary information on the chrY. The Y-ARS represents the MRCA of almost all human paternal lineages known so far and improves capturing evolutionarily derived alleles, making it a valuable resource for various evolutionary genetics applications, such as TMRCA estimation, phylogenetic analyses and demographic modeling. We further provide a publicly available tool, polaryzer, that utilizes the information in the Y-ARS to determine whether SNPs reported in VCF files (after alignment to GRCh37, GRCh38, T2T) are ancestral or derived. Further, we share the resources of generating this Y-ARS, to be extended on other species as well.

## Materials and Methods

### Ancestral State Reconstruction

**Fig. 4.**
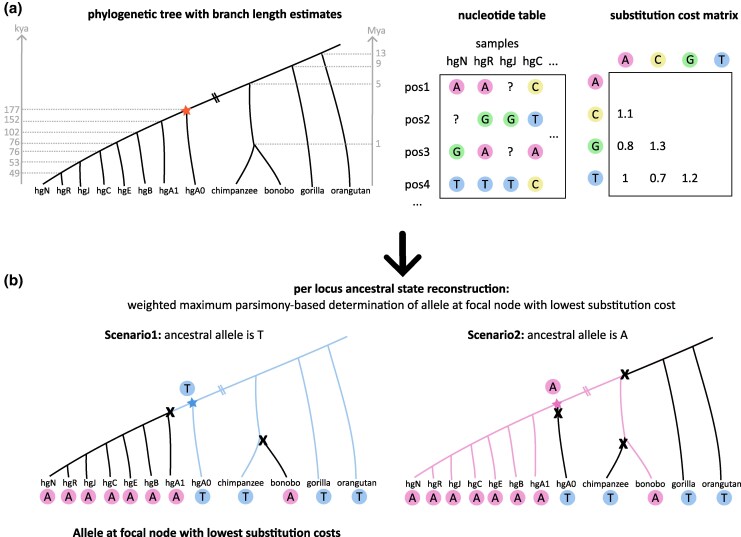
WMP approach for ASR per locus. a, left) Phylogenetic relationships of orangutan, gorilla, bonobo, chimpanzee and the human haplogroups A0, A1, B, C, E, J (T2T-CHM13), N, R. The focal node, i.e. the MRCA of most known paternal lineages, is marked with a star. Lineage split times are presented in gray in thousand (kya) and million years (Mya). Other inputs include the (a, middle) nucleotide table of all polymorphic sites and (a, right) defined nucleotide substitution costs. b) An example for allele cost calculation with alleles T (scenario 1) and A (scenario 2) as potential focal node alleles, where the costs are calculated by dividing the nucleotide substitution cost by the branch length factor. The scenario with the lowest total cost (here: allele T) is the estimated ancestral allele at the focal node.

In the following, we aimed to approximate the Y-ARS—the MRCA of most known human chrY sequences—by reconstructing the ancestral state at each Y-chromosomal site (Fig. 4). To reconstruct the ancestral allele at a node of interest (i.e. focal node) it is necessary to combine (A) alleles at the same genomic position of the investigated samples, (B) the samples' phylogenetic relationships, and (C) apply an evolutionary model suitable for the data.

### Identifying the Variable Sites Among chrY Sequences

To generate the Y-ARS, we selected the T2T-CHM13-v.2.0 Y chromosome sequence (T2T-Y) (haplogroup J1a2a1a2c1a1) as a template to maintain an already established coordinate system. Ancestral alleles were reconstructed at the sites along the T2T-Y template sequence, using long-read HiFi sequencing data of seven human samples of haplogroups A0b, A1a, B2b, C1a, E1b, N1a, R1b (Supplementary table S6, Supplementary Material online). The samples were processed according to the long-read sequencing data processing pipeline shown on Github (see https://github.com/ZehraKoksal/Y-ARS/tree/main/Commands). In short, all FASTQ files per sample were merged and then aligned to the T2T reference using minimap2 (-ax map-hifi -*P* 0.95 –secondary = yes -N 1 -a -L -eqx –MD) (Li 2018). Resulting BAM files were sorted using samtools v1.18 (Li et al. 2009), and variants were called only for the chrY using freebayes v0.9.21 (-*P* 1 –min-mapping-quality 50 –min-base-quality 20 –no-indels –no-mnps –no-complex) (Garrison and Marth 2012). After identifying polymorphic positions within all samples, these 14,528 target positions were re-called for all samples (−report-monomorphic). The variants were filtered by keeping only sites with phred-scaled quality score QUAL ≥ 20 within X-degenerate regions (XDR), X-transposed regions (XTR), and ampliconic regions (AMP). We removed loci located within inverted repeats (IRs), arms of the palindrome regions 1 to 8 (P1-8) and the testis specific protein Y-linked (TSPY) repeat arrays (Rhie et al. 2022) (Supplementary tables S7 and S8, Supplementary Material online), which yielded a total of 11,535 polymorphic sites used for ASR.

In the next step, we assessed 9,395 out of the 11,535 sites polymorphic among the human sequences in primate sequences (2,140 loci were excluded only for the primate data due to their location on the human-specific XTR). Y chromosome sequences from chimpanzee, bonobo, gorilla, and orangutan genome assemblies (Supplementary table S6, Supplementary Material online) from Makova et al. (2024) were downloaded as FASTA files. The FASTA files were aligned to the T2T reference sequence using minimap2, and the resulting BAM files were sorted and indexed. Variable and nonvariable alleles were called at the 9,395 predefined sites using BCFtools v1.9 (Li 2011) commands “mpileup”, “call”, and “filter” to call SNPs with minimum mapping quality ≥ 50, minimum base quality ≥ 20, and read depth > 2 (bcftools mpileup -Ou -q 50 -Q 20 | bcftools call –ploidy 1). The commands are shared in the accompanying Github repository.

### Phylogenetic Relationships of Samples and Substitution Cost Matrix

The phylogenetic relationships and lineage divergence time estimates for the human samples were taken from Hallast et al. (2023) and for the primates from Kivell (2019) (Fig. 1, Supplementary fig. S11, Supplementary Material online). To avoid a bias in favoring mutational events on longer branches, the branch lengths were normalized to the shortest branch having a scaled length of 1. This was done by taking the decadic logarithm of the branch age estimates and increasing this value by 6. We further incorporated a simple nucleotide substitution cost matrix in the ASR, which is a modified substitution model of the Kimura-2-parameter model (Kimura 1980) with symmetrical and ranked substitution costs favoring transitions over transversions.

### WMP Approach for Reconstructing Ancestral States

To construct the Y-ARS, we used a WMP approach to estimate ancestral alleles at the 11,535 loci. We combined the information of the alleles observed along the samples' sequences (nucleotide matrix) together with the phylogenetic tree including branch lengths and a substitution cost matrix (Fig. 4). Using this information, we created a custom python script (shared on Github) to calculate the most parsimonious allele for the focal node, i.e. the MRCA of most known paternal lineages. At each locus, the allele with the lowest mutational costs at the focal node was selected as the most parsimonious allele (i.e. most likely ancestral allele).

### The Ancestral-like Y-chromosomal Reference Sequence

After determining the most likely ancestral alleles, we converted all derived SNPs on the T2T-Y FASTA sequence back to ancestral states (*N* = 2,545) using the SeqIO module from the Bio package v1.84 in Python v3.11.11. We replaced the chrY in the T2T reference with the Y-ARS, keeping all other chromosomes in the reference unaltered.

To estimate the age of the Y-ARS (i.e. focal node), we used a rate of 8.71 × 10^−10^ (CI 9.43 × 10^−10^ to 8.03 × 10^−10^) mutations per position per year (Helgason et al. 2015), number of positions at nonrepetitive regions of the chrY (15,588,924 bp), and the number of derived sites on the T2T sequence (*N* = 2,545).

### Annotating the Y-ARS Using YBrowse Information

To assess if the converted SNPs match with existing database annotations, we downloaded haplogroup annotations from the Human Y Chromosome Pangenome Browser “YBrowse” (https://ybrowse.org/; version update 14.02.2025), which compiles information from major chrY databases as well as single submissions by researchers. The downloaded SNPs were filtered by removing SNPs with unknown YCC or ISOGG haplogroup annotations and SNPs annotated at position 1 or at chromosomes other than “chrY”, yielding in 1,783,937 SNPs with a haplogroup annotation (including sites with multiple annotations).

### Examining the Y-ARS for Haplogroup A0 Bias

Due to the unique location of the A0 branch relative to the MRCA of all humans (i.e. the focal node) in the phylogenetic tree, SNPs exclusive to haplogroup A0 might be biased in the absence of any primate data despite a thorough ASR. To investigate for bias toward possible SNPs exclusive to haplogroup A0, we further investigated the 2,520 positions with an allele specific to A0 among all human samples by accessing these positions in two of the 40 human short-read sequencing data of haplogroup A0 (HG01890 and HG02982) (Supplementary table S9, Supplementary Material online) and two additional haplogroup A00 samples GRC13292545 and GRC13292546 taken from Karmin et al. (2015). The haplogroup A00 samples were downloaded as GRCh37-aligned BAM files and therefore converted to FASTQ files (samtools fastq GRC13292546.chrY.bam −1 r1_GRC13292546.fastq −2 r2_GRC13292546.fastq -s singles_GRC13292546.fastq −0/dev/null -n) prior to following the short-read sequencing pipeline and alignment to the T2T reference sequence. The resulting BAM files of the two haplogroup A00 samples were merged to account for the low coverage (∼0.25× less reads aligned to chrY) compared to the HgA0 samples. The alleles at the positions of interest were extracted from the generated BAM files using BCFtools (bcftools mpileup -Ou -q 50 -Q 20 -R 2520_sites.csv -f T2T.fasta sample.bam | bcftools call –ploidy 1 -O v -o out_sites.vcf -c; bcftools filter -e “TYPE=‘INDEL’” out_sites.vcf -o sites_noindels.vcf). Additionally, the chrY FASTA sequences of the three primate reference sequences, PanTro3 (NC_072422.2, NHGRI_mPanTro3-v2.0), PanTro6 (NC_006492.4, Clint_PTRv2), and PanPan1 (NC_073273.2, NHGRI_mPanPan1-v2.0_pri) were accessed and aligned to the T2T reference sequence using minimap2. In the sorted and indexed BAM file, “bcftools mpileup” was used to extract the alleles at defined sites using the above command. The commands are shared in the accompanying Github repository.

### Quantifying Reliability of Y-ARS Alleles Using Uncertainty Score

To quantify how reliable the ancestral alleles are at all 11,535 sites along the Y-ARS with reconstructed alleles, we determined an uncertainty score for each of the 11,535 alleles (Supplementary table S3, Supplementary Material online). The per-locus uncertainty score of a reconstructed ancestral allele is defined by the following formula:


$$uncertainty\;score_{locus}=\frac{\;M}{s},\;\mathrm{where}\;1\leq M\leq 6\;\mathrm{and}\;2\leq s\leq 12$$

and where *M* equals the number of mutational events required to explain the observed alleles during ASR and *s* equals the total number of samples with available allele information during ASR. The resulting uncertainty score can range from 1/12 to 1/2 with a lower score corresponding to a more reliable annotation of the Y-ARS allele as “ancestral”. For the example in Scenario 1 of Fig. 4b, an uncertainty score of 2/12 was determined.

### Benchmarking the Y-ARS Using 40 Short-read Sequences Across Different Haplogroups

To evaluate the performance of the Y-ARS, we assessed the alignment and variant calling of 40 short-read sequencing male samples from the 1000 Genomes Project (1000 Genomes Project Consortium et al. 2015; Fairley et al. 2020) with this new reference. The samples were representative of most major haplogroup clades from A0 to T (Supplementary table S9, Supplementary Material online). We utilized paired-end data of low coverage WGS from the GRCh38 data collection, as the latest data collection of unaligned sequences in FASTQ format. After trimming of the FASTQ files using trimmomatic v0.39 (Bolger et al. 2014), the reads were aligned to Y-ARS and existing references GRCh37, GRCh38, T2T-CHM13-v.2.0 using bwa v07.7.17 mem (Li and Durbin 2009), sorted and merged using samtools. Upon addition of read groups, PCR duplicates were removed using picard MarkDuplicate v2.26.4 (Broad Institute 2019). The resulting BAM files were indexed using samtools and base quality scores were recalibrated using Genome Analysis Toolkit (GATK) v4.2.0.0 BaseRecalibrator and ApplyBQSR (Van der Auwera and O’Connor 2020). Genetic variants were identified using BCFtools commands “mpileup”, “call” and “filter” to call SNPs with minimum mapping quality ≥ 50 and minimum base quality ≥ 20 (bcftools mpileup -Ou -q 50 -Q 20 | bcftools call –ploidy 1). The variants were filtered to exclude indels and sites with depth <2 and sites falling outside the well-mappable area on the chrY (Poznik et al. 2013). Details on the commands used for data processing are shared on Github.

To compare the called positions, all coordinates were lifted to T2T with R-package rtracklayer (Lawrence et al. 2009) in R v.4.1.2 (R Core Team 2021). We used ISOGG (v.15.73) (https://isogg.org/tree/) and YFull (v10.01) (https://www.yfull.com/tree/) haplogroup annotations for annotating the sample-specific SNPs. The phylogenetic relationships among the 40 samples were generated for visualization purposes using the established haplogroup phylogenies reported in the ISOGG database by applying Y-LineageTracker (Chen et al. 2021).

### Normalization of SNP Counts and Age Estimation

Since the samples had differing coverages (Supplementary fig. S12, Supplementary Material online), we normalized the SNP counts to compare these across samples by dividing the raw SNP count by coverage and multiplying this by 10,000,000 to estimate the SNP counts per 10 million base pairs.

We further calculated the age of split for each sample from the reference sequences GRCh37, GRCh38 and T2T by the following equation:


$$Time\;of\;split\;=\frac{derived\;SNPs}{coverage\;*\;mu}$$

where the *derived SNPs* correspond to the number of evolutionarily derived SNPs, *mu* to the mutation rate 8.71 × 10^−10^ (CI 9.43 × 10^−10^ to 8.03 × 10^−10^) mutations per position per year (Helgason et al. 2015) and *coverage* to the number of positions with at least 2× coverage on chrY region of interest (10.5 Mb).

### Determining Ancestral and Derived Alleles in VCF Files Using Polaryzer

Polaryzer v1.1 (https://github.com/ZehraKoksal/Y-ARS/tree/main/Polaryzer) was run using the VCF files of all 40 samples (generated using variant calling thresholds: read depth ≥ 2 and excluding indels). The VCF files generated upon alignment to GRCh37 and GRCh38 were lifted over to reference T2T using rtracklayer in R v.4.1.2 and then used as input for polaryzer after filtered for nonrepetitive regions. The specific commands used are available in the Github repository. To illustrate the fraction of ancestral and derived alleles per sample and reference sequence, barplots were generated using seaborn v0.13.2 in python v3.11.11.

## Supplementary Material

msaf222_Supplementary_Data

## Data Availability

All 1000 Genomes Project population samples used in the analyses are available from the NHGRI Repository at Coriell. Other sequences are available at the European Nucleotide Archive (long-read human: PRJEB58376), at the National Center for Biotechnology Information NCBI (primate assemblies: PRJNA930783, PRJNA930872, PRJNA930872, PRJNA930875) and at the data repository of the Estonian Biocentre (http://www.ebc.ee/free_data/chrY) (short-read human haplogroup A00 samples: GRC13292545 and GRC13292546). Code for generating the Y-ARS v1.1 is available on Github (https://github.com/ZehraKoksal/Y-ARS). The polaryzer v1.1 tool for determining the allelic state of variants reported in VCF files is available on Github (https://github.com/ZehraKoksal/Y-ARS/tree/main/Polaryzer). The Y-ARS v1.1 is available on zenodo (https://doi.org/10.5281/zenodo.15723730).
