# Supplementary material for: Introducing the Y-chromosomal Ancestral-like Reference Sequence—Improving the Capture of Human Evolutionary Information
Source: Mol Biol Evol. 2025 Sep 12;42(10):msaf222. doi: 10.1093/molbev/msaf222 (PMC12485614; doi:10.1093/molbev/msaf222)
Supplement: msaf222_Supplementary_Data [file msaf222_supplementary_data.zip › Y-ARS_SupplementaryFigures_revision.pdf]

Supplementary figures

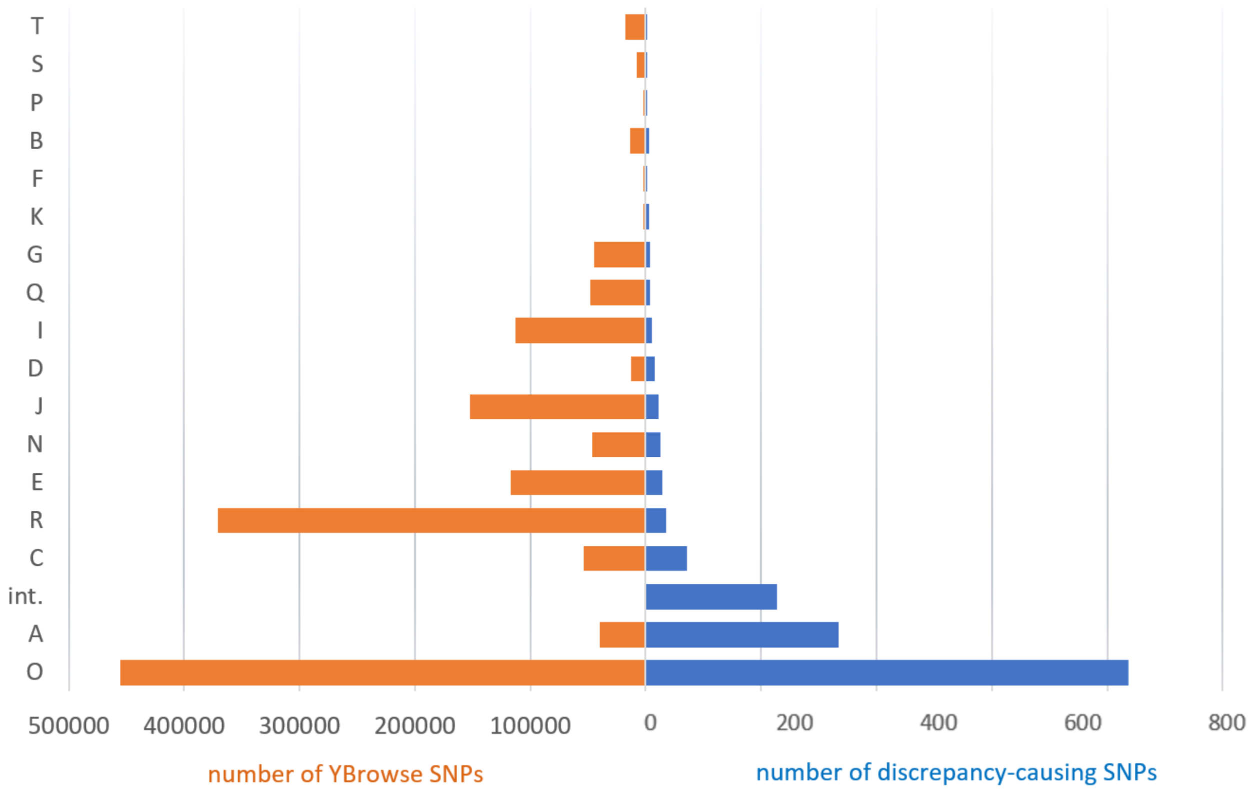

Fig. S1 Assessment of haplogroup annotations from the YBrowse database across the entire length of the Y-ARS. Overall reported haplogroup-defining SNPs in YBrowse are shown in orange (N=1,499,841), while SNPs that conflict with the ancestral allele defined in the Y-ARS sequence are shown in blue (total=1,311 out of 1,483 SNPs with haplogroup annotation). Annotation "int." comprises intermediate haplogroups BT, CT and CF. Most discrepancies were found for SNPs defining haplogroup O, which also has the most annotations in the database. Second-most discrepancies are caused by haplogroup A, possibly indicating inaccurate entries in the database due to the location of the haplogroup close to the focal node.

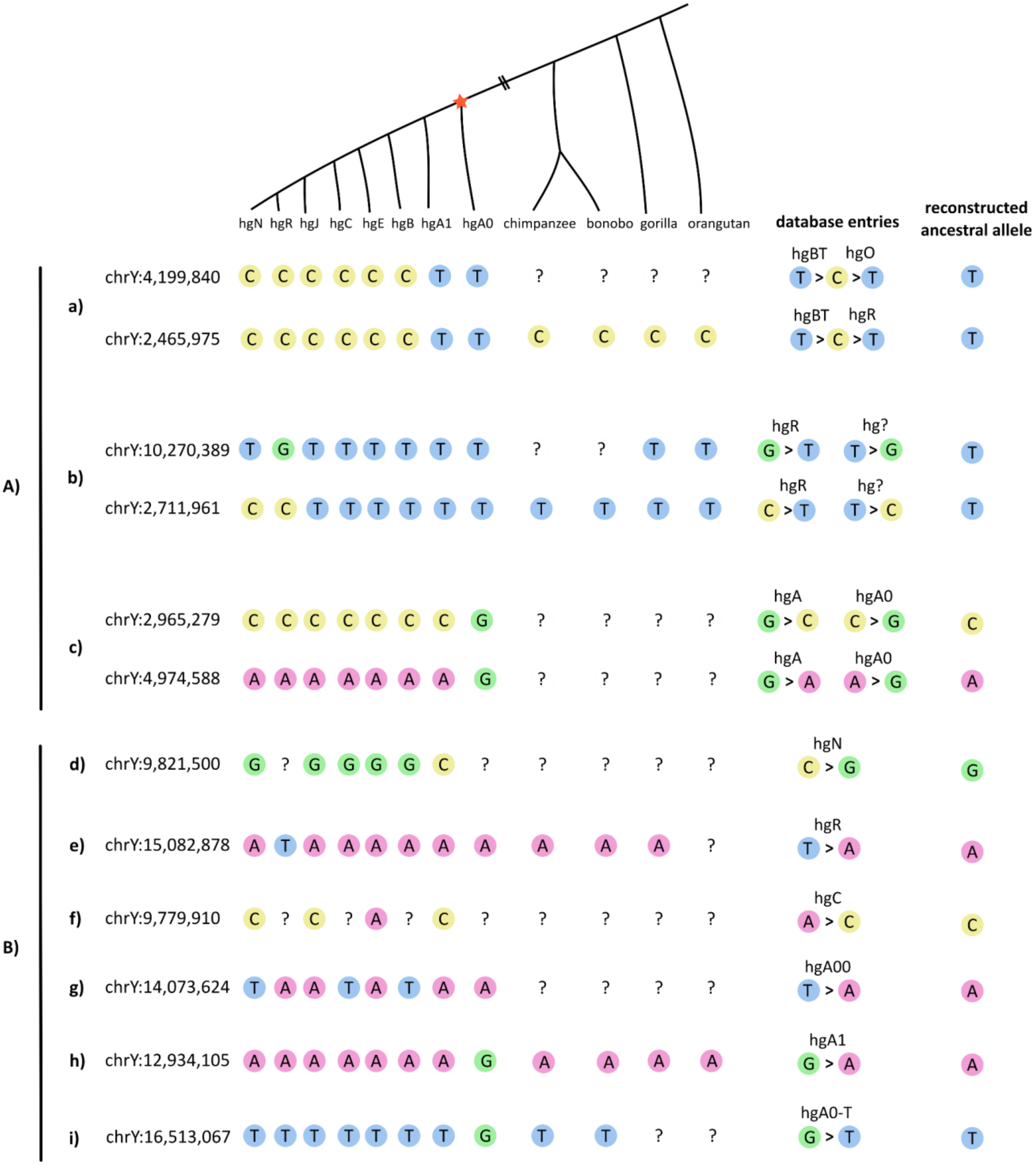

Fig. S2 Allele comparisons between Y-ARS and YBrowse database at positions with conflicting ancestral allele annotations. The observations are divided into cases where A) the contradiction is caused by multiple entries in the database, and where B) the YBrowse derived allele does not match the Y-ARS allele. The contradictions between the database and the Y-ARS can be due to

(a) back-mutations, (b, e) wrong database entries that likely resulted from using reference sequences of downstream haplogroups, (c, h, i) discrepancies in upstream haplogroups or inaccurate database entries, (d, f) high rates of missingness, and (g) high mutability at genetic loci.

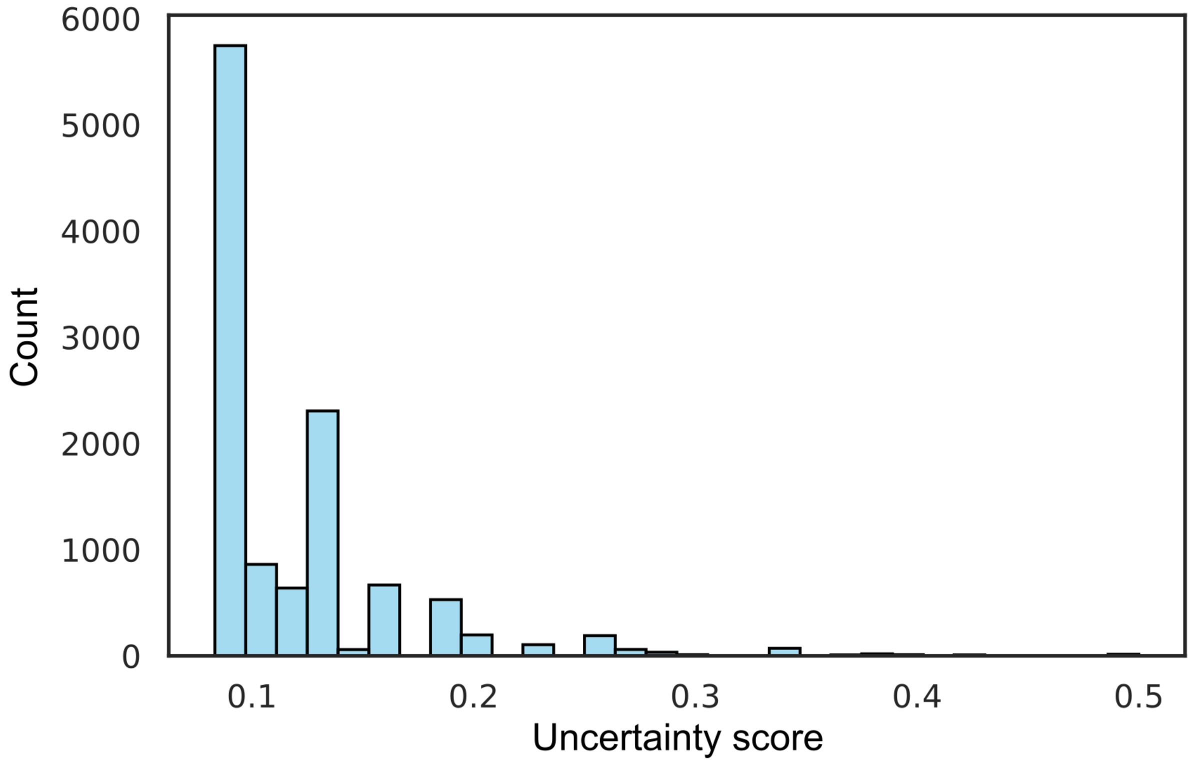

Fig. S3: Distribution of uncertainty score per site across all 11,535 reconstructed alleles on the Y-ARS. The uncertainty score is defined as the number of mutational events required to explain the observed allele divided by the total number of samples with non-missing data during ASR. A lower score corresponds to a more reliable annotation of the site as ancestral.

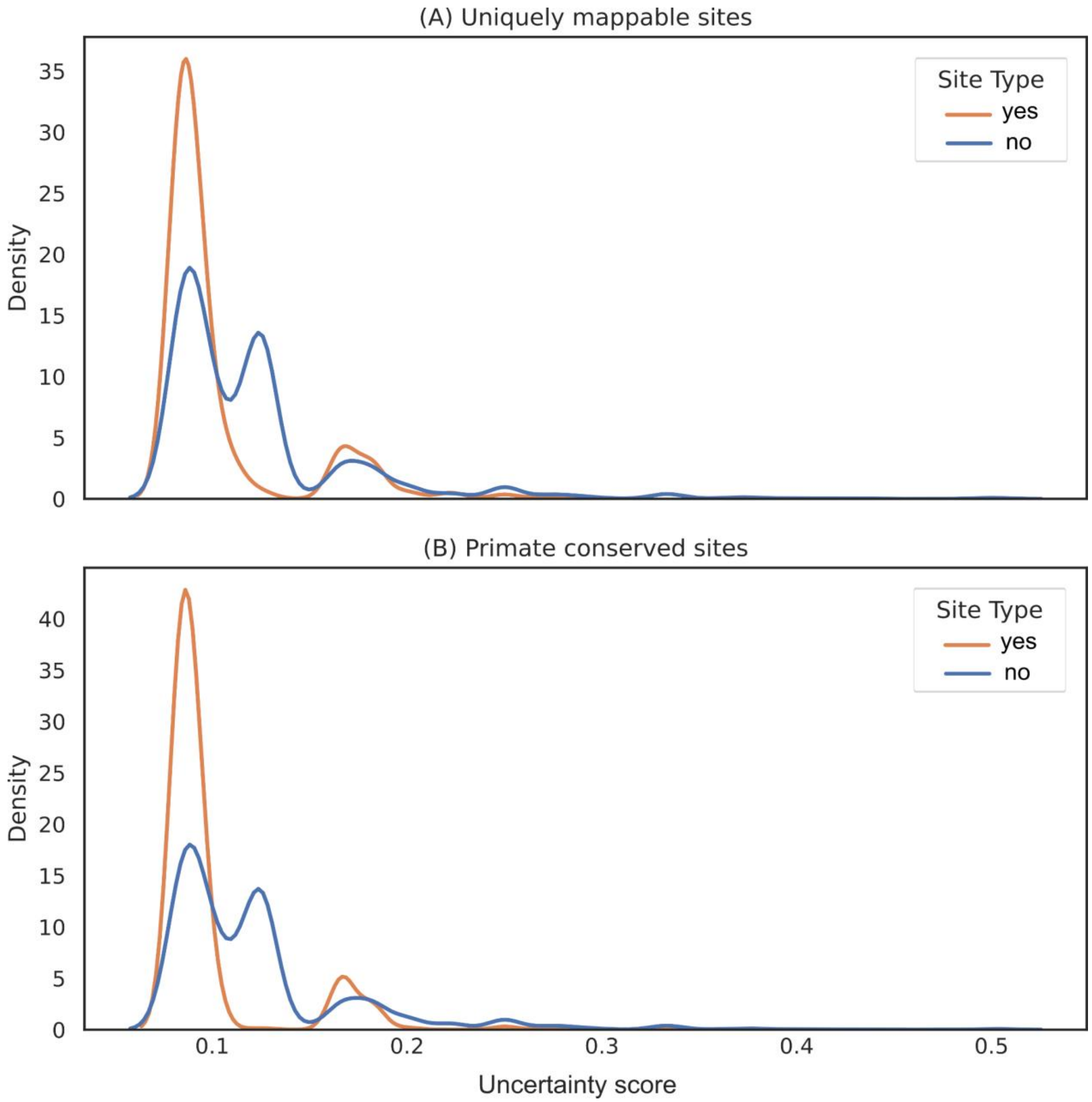

Fig. S4: Comparison of the uncertainty scores on the 11,535 Y-ARS sites with reconstructed alleles between sites falling into (orange) and outside (blue) different sequence classifications from Swiel et al. (2025). Sites classified as A) uniquely mappable on the chrY, or B) conserved between human and primate (both presented in orange) show an enrichment for lower uncertainty scores compared to sites not falling into these classifications (as shown in blue). Uncertainty scores of the ancestral alleles on uniquely mappable regions were on average 0.103 (±0.036 SD; median:0.091) and outside these regions 0.122 (±0.052 SD; median:0.111). On primate-

conserved regions, the uncertainty scores were on average 0.099 (±0.032 SD; median:0.091) and outside of primate-conserved regions 0.123 (±0.052 SD; median:0.111). The density values on the y-axis are normalized within each group to a total per-group under the curve area of 1.

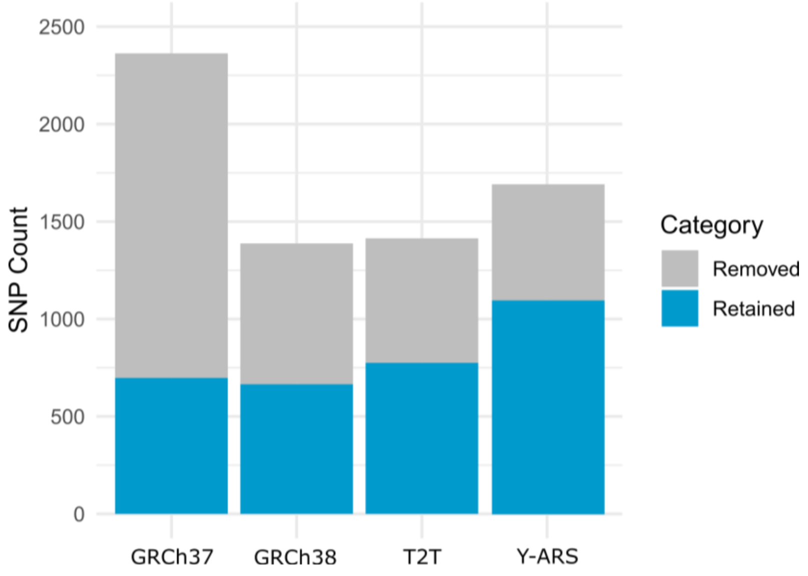

Fig. S5 Average SNP count before and after quality control of the 40 short-read samples used for benchmarking analysis aligned to each of the reference sequences. Gray color indicates removed sites falling into repetitive and poorly-mappable regions defined by Poznik et al. (2013), while blue color indicates SNPs on well-mappable regions (see Fig. 1). Before quality control, GRCh37 alignments resulted in calling the highest number of SNPs on average across samples caused by excess read mapping on non-informative regions of the Y chromosome, such the centromere and Yq heterochromatic arm.

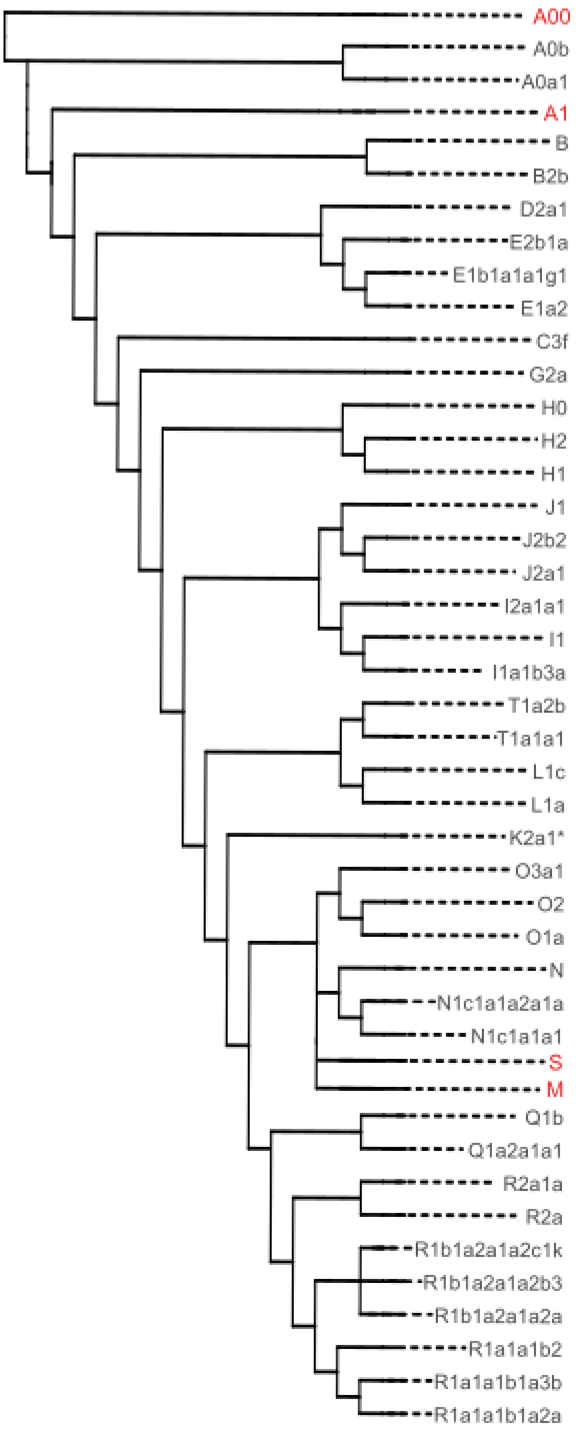

Fig. S6 Phylogenetic relationships of the 40 short-read samples used for benchmarking (black), and the location of missing major haplogroup lineages not included in the analyses (red). The branch lengths are unscaled and do not reflect evolutionary time.

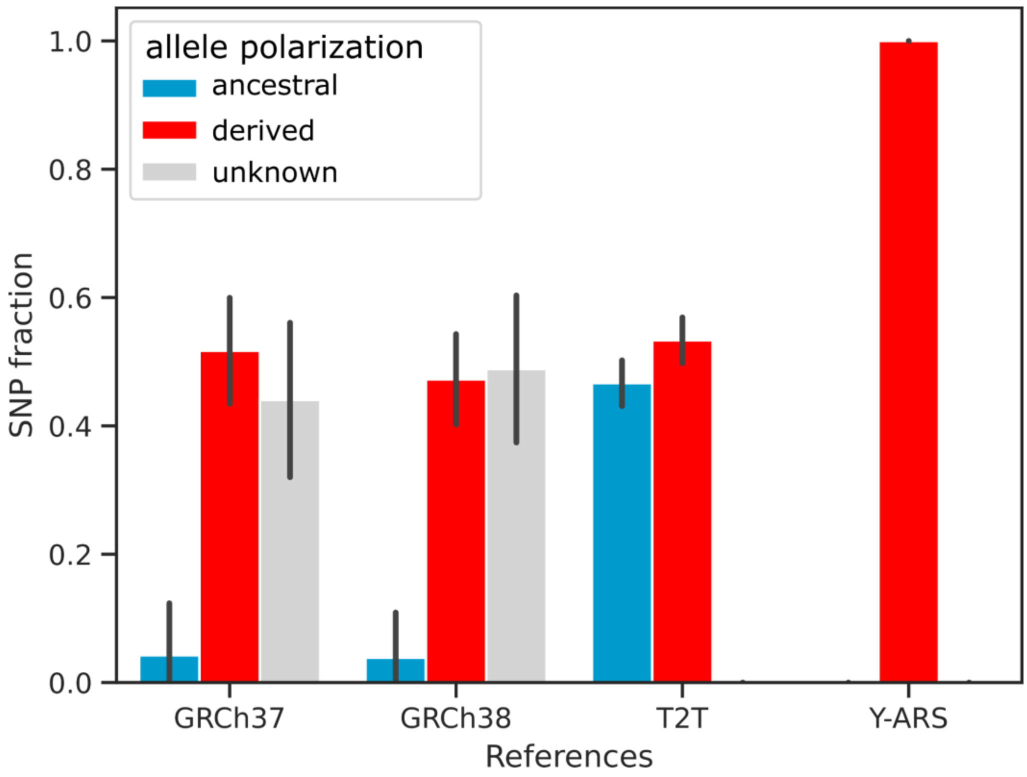

Fig. S7 Comparison of allele polarization for variants called after alignment to different reference sequences. The bar height presents the average fraction of SNPs annotated as ancestral (blue), derived (red) and unknown (gray) over all 40 samples, and the error bars indicate the standard deviation. Annotating variants in GRCh37/GRCh38 coordinates contains alleles with missing annotation (44%-49%), caused by lifting over variants from Y-ARS (T2T coordinates) to GRCh37 or GRCh38. The variants with unknown allelic states can be filtered out manually or by lifting over GRCh37/GRCh38 coordinates to T2T prior to using polaryzer.

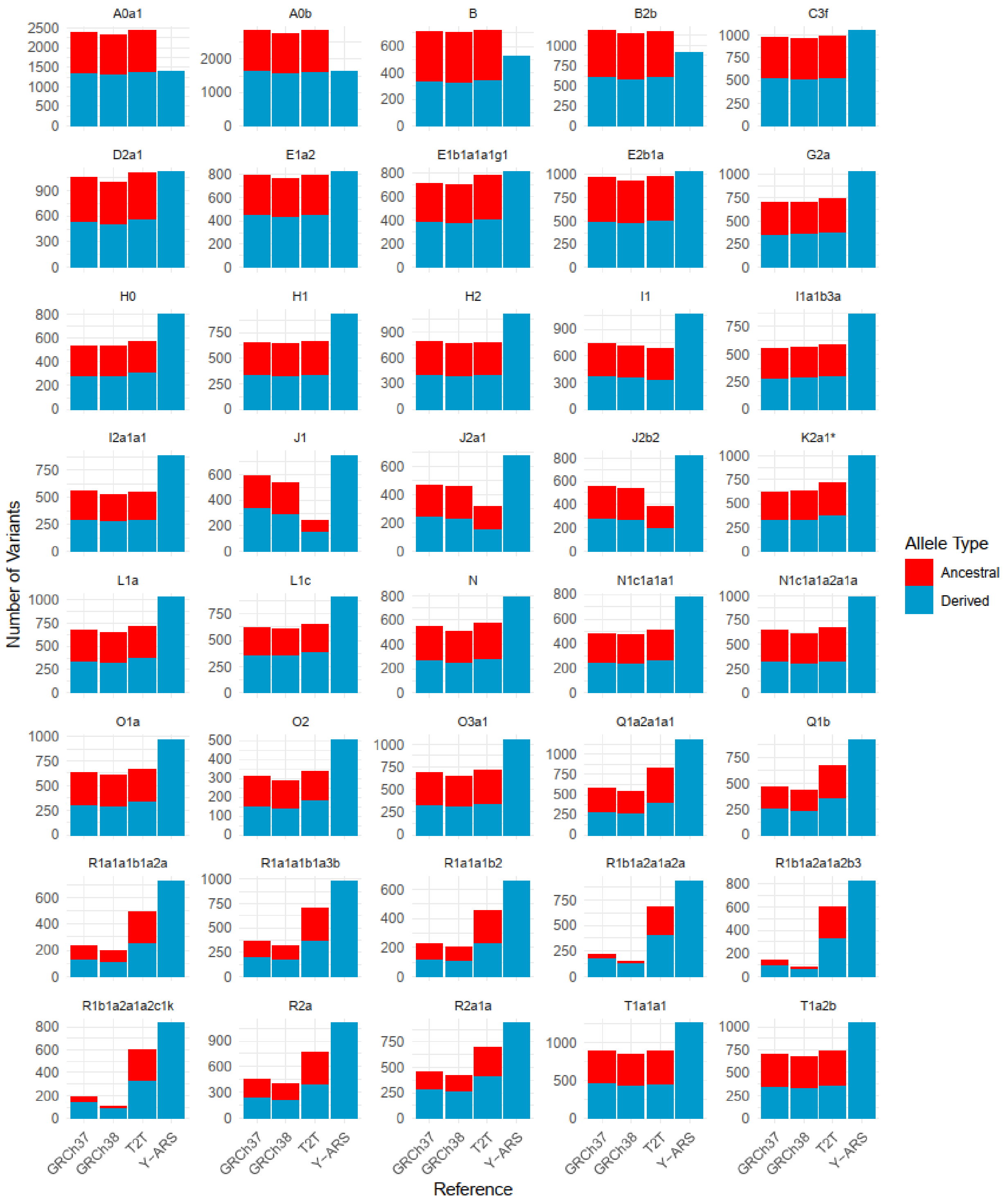

Fig. S8 Number of variants annotated as evolutionarily ancestral and derived (using polaryzer) across the 40 short-read samples and all four references.

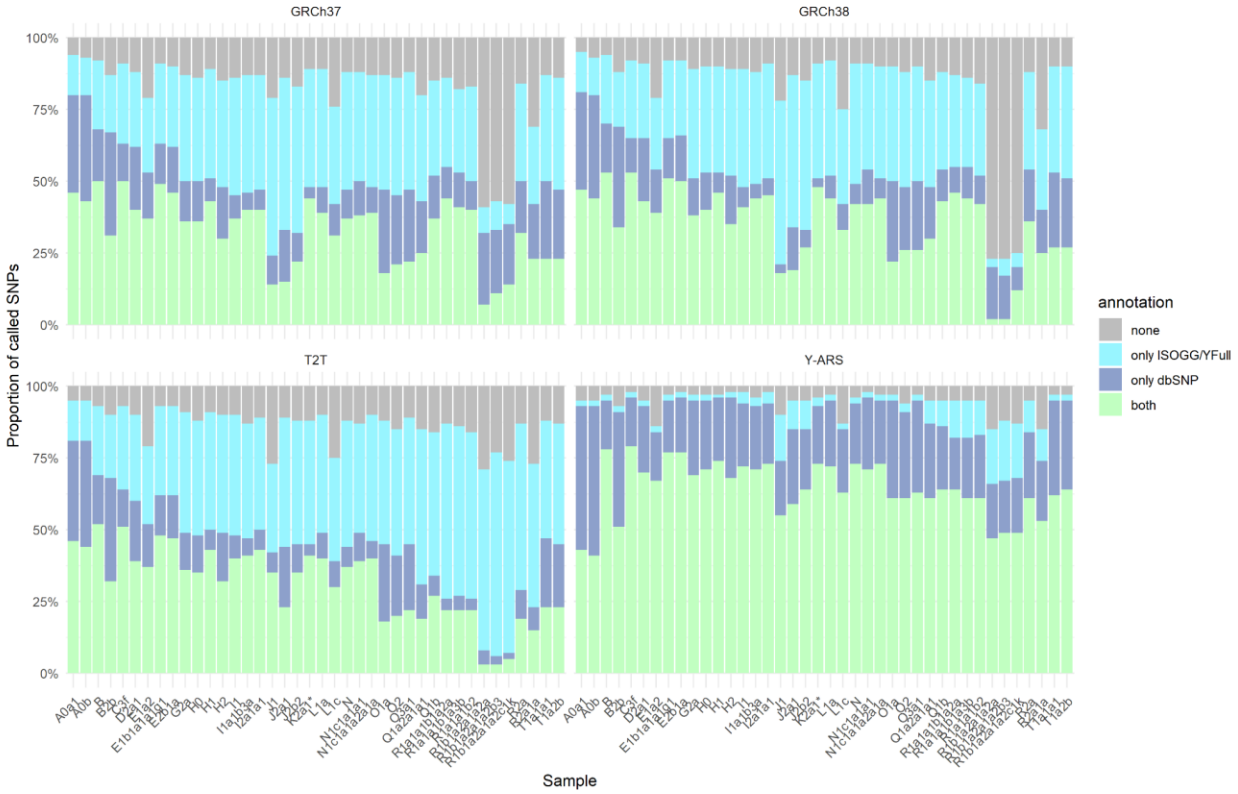

Fig. S9 Proportion of SNPs that are not annotated or that have annotations in dbSNP or/and ISOGG/YFull databases in the 40 short-read sequences after alignment to the four reference sequences.

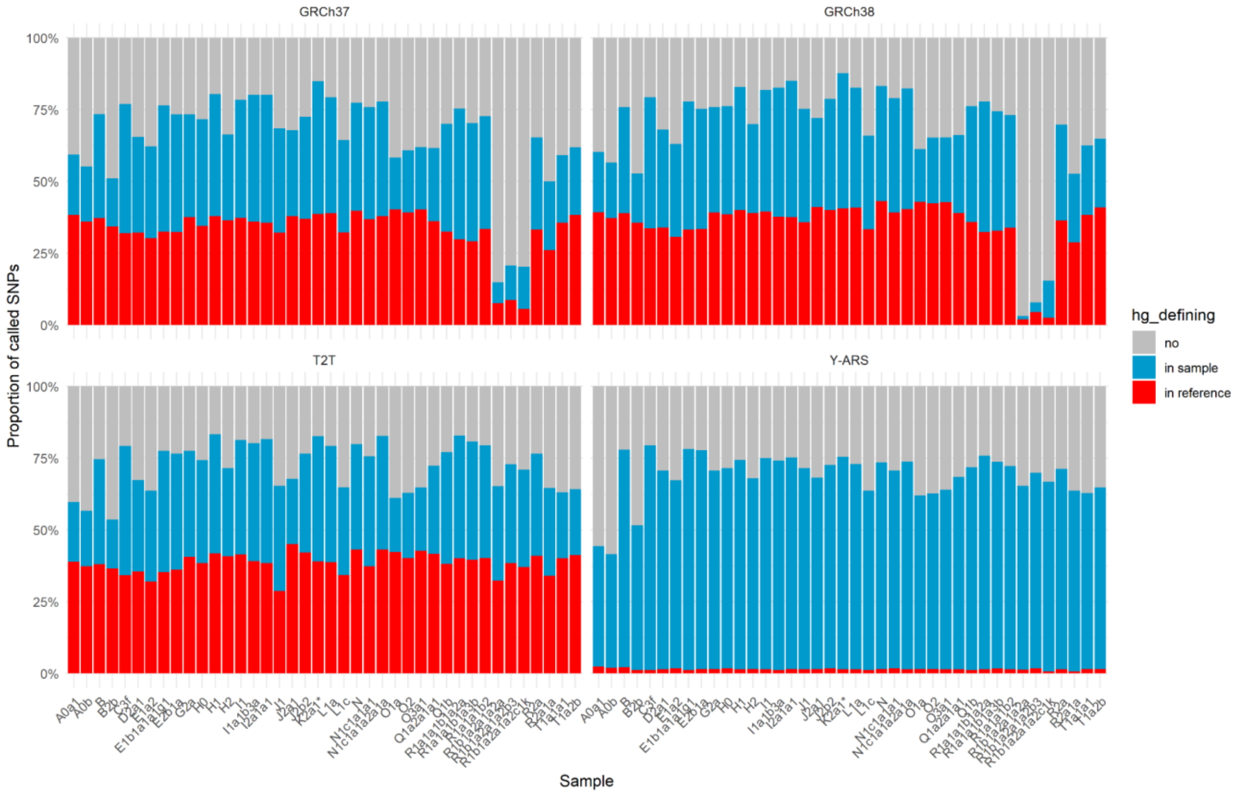

Fig. S10 Proportion of SNPs that carry the haplogroup-defining allele (from ISOGG/YFull database) across the 40 short-read samples and all four references. Sites where the haplogroup-defining allele is present in the sample are shown in blue, whereas sites where the haplogroup-defining allele is present in the reference sequence are shown in red. Gray indicates SNPs without known haplogroup annotations. After alignment to GRCh37, GRCh38 and T2T, approximately 53% of the haplogroup-defining SNPs show the haplogroup-defining allele in the sample sequence, while 47% show the haplogroup-defining allele in the reference sequence.

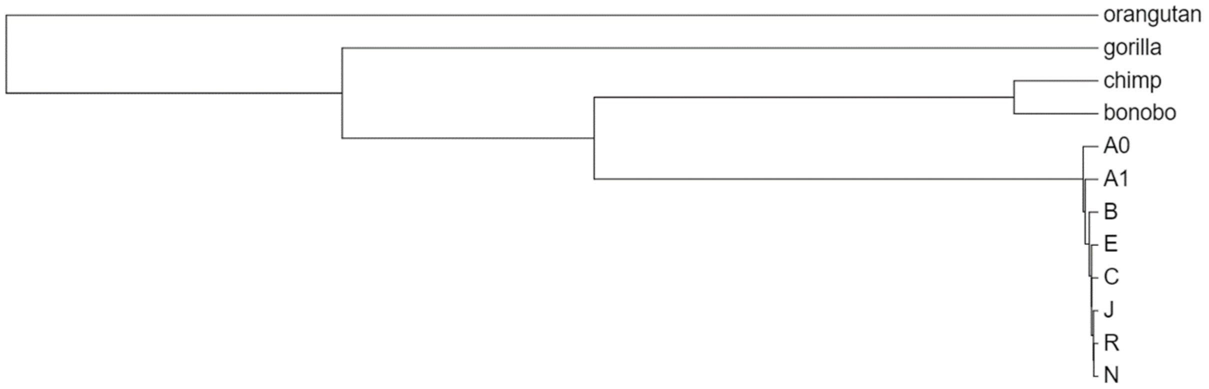

Fig. S11 Phylogenetic tree of eight human and four primate samples used for ancestral state reconstruction taken from (Hallast et al. 2023) and (Kivell 2019).

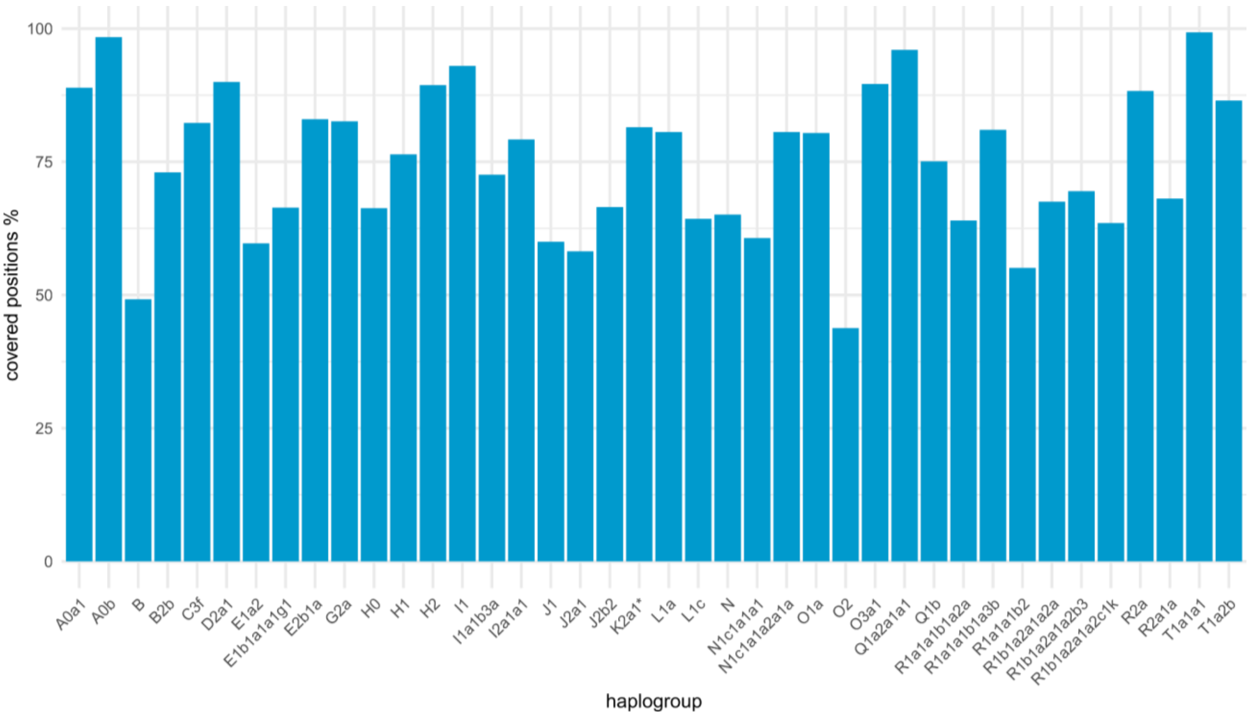

Fig. S12 Fraction of positions relevant to T2T reference covered with at least 2 reads (2x) within regions accessible by short-read sequencing as defined by Poznik et al. (2013) for each of the 40 short-read samples.
